# Genetic diversity of the recovered populations of *Mesocriconema xenoplax* (Nematoda: Criconematidae) from orchards in Fars province, Southern Iran

**DOI:** 10.2478/jofnem-2024-0048

**Published:** 2024-12-24

**Authors:** Ali Asghar Dehghan, Reza Ghaderi, Akbar Karegar, Abbas Mokaram Hesar

**Affiliations:** Department of Plant Protection, School of Agriculture, Shiraz University, 71441-65186, Shiraz, Iran; Department of Plant Protection, Faculty of Agriculture, Afagh Higher Education Institute, Urmia 5756151818, Iran

**Keywords:** Genetic diversity, haplotype, ISSR, morphometrics, phylogeny, ring nematode

## Abstract

In this survey, 14 populations of *Mesocriconema xenoplax* were collected from the rhizosphere of eight fruit and nut trees in Fars province, Southern Iran. The phylogenetic relationships of these populations with other representatives of the species were investigated using sequences of cytochrome c oxidase subunit 1 mitochondrial gene (*COI*) and D2-D3 expansion fragments of 28S rDNA. Phylogenetic studies indicated a close relationship of the currently sequenced populations with known haplotype groups (HG) in the *COI* tree and revealed two separate lineages in the 28S rDNA tree. Moreover, the genetic diversity of the populations was analyzed using seven ISSR primers as molecular markers. The estimated genetic diversity among populations regarding associated trees and geographic regions were low values of 3.3% and 5.9%, respectively, indicating high gene flow among the recovered nematode populations. On the other hand, the estimated fixation index (F_ST_) was higher for associated plants than for geographic regions (0.611 *vs* 0.504) indicating that plant-based population segregation better explains genetic diversity in this species. This work expands our knowledge of the genetic structure of this cosmopolitan species of plant-parasitic nematodes.

The ring nematode, *Mesocriconema xenoplax* (Raski, 1952; Loof and De Grisse, 1989), is a cosmopolitan species (Peneva et al., 2000; Wouts, 2006; Andrássy, 2007; Geraert, 2010; Powers et al., 2014), parasitic on numerous perennial crops (Pinkerton et al., 1999), especially grapevine (Orton Williams, 1972; Klingler and Gerber, 1972; Klingler, 1975; Ambrogioni et al., 1980; McKenry, 1992; Ramsdell et al., 1996; Pinkerton et al., 1999; Pinkerton et al., 2004) and peach (Lownsbery et al., 1973; Nyczepir et al., 1983; 1987; 1988).

The degree of genetic diversity among different populations of a given species (e.g., *M. xenoplax*) is highly dependent on the events that control its evolution, such as gene flow, mutation, genetic drift, natural selection, etc., which occur independently in each population (Lax et al., 2007; Mokaram Hesar et al., 2022). Various approaches are used to study genetic variation between populations of nematodes with neutral molecular markers including Inter Simple Sequence Repeats (ISSR) and Random Amplified Polymorphic DNA (RAPD) are well known. Both techniques can provide comparable quantitative results (Lax et al., 2007; Tigano et al., 2010; Mokaram Hesar et al., 2022) with ISSR being more reproducible compared to RAPD (Lax et al., 2007). ISSR markers use a quantitative analysis of population differentiation (diversity) based on certain population statistics such as fixation index (F_ST_) and indirect gene flow (N_m_) (Wright, 1965) and, alternatively, by the genetic differentiation index usually referred to as Nei’s genetic diversity (Nei, 1973; Slatkin and Barton, 1989). Allele frequency is also calculated as the number of times an allele appears within sampled homozygous and heterozygous individuals divided by twice the number of individuals sampled. The Hardy–Weinberg equation allows the conversion of allele frequencies into genotype frequencies (Zhou et al., 2009).

A few studies have investigated the intraspecific diversity of *M. xenoplax* using morphological characters (Cordero et al., 2012) or haplotype-based phylogenetic analyses (Powers et al., 2014). The first group provided morphometric data for 16 populations of *M. xenoplax* collected from various localities in the USA from both herbaceous and woody plants, and the second group found 24 HG in the genus *Mesocriconema* including seven well-supported subgroups (HG from 8 to 14) in *M. xenoplax* based on phylogenetic analysis of *COI* that had mean genetic distances of 2.7%–12.8%. Morphological and morphometric characteristics of the species have been presented for different populations from diverse plants and regions in Iran (Loof and Barooti, 1991; Karegar et al., 1995; Jamali et al., 2004; Aliramaji et al., 2006; Eskandari et al., 2007; Gharakhani et al., 2007; Chenari et al., 2009; Deimi and Mitkowski, 2010) which are documented in Eskandari (2018), but the genetic intraspecific diversity of the species using ISSR markers has yet not been explored.

The present study aims to investigate the molecular diversity of different populations of *M. xenoplax* recovered from the rhizosphere of fruit and nut trees in Fars province, Southern Iran, using reconstructed phylogenetic trees and ISSR markers. Quantitative analyses based on the ISSR technique were implemented to discover the amount of gene flow (exchange) and genetic diversity within and between populations of *M. xenoplax* regarding associated plants and geographic regions.

## Materials and Methods

### Samplings and morphological characterization of the recovered populations

A total of 14 populations of *Mesocriconema xenoplax* were collected in 2018–2019 from the rhizosphere of fruit and nut trees including walnut (five populations), grapevine (two populations), almond (two populations), apricot, apple, peach, sour cherry and greengage from several localities in Fars province, Southern Iran (Table 1). Soil composite samples were prepared by taking soil from three cores around the trunk of 5–10 trees in each orchard. Nematode specimens were extracted from the soil using the centrifugal flotation method (Jenkins, 1964), killed and fixed with hot FPG (4:1:1, formaldehyde: propionic acid: glycerol), and processed to anhydrous glycerol (de Grisse, 1969). They were then mounted in glycerol on permanent slides using paraffin wax and were studied using an Olympus BX41 light microscope equipped with a Dino-eye microscope eye-piece camera and its Dino Capture version 2.0 software. For each population, the nematodes were examined and photographed, and 10 females were measured using the same microscope. All populations were characterized based on morphological and morphometric characters using identification keys (e.g., Geraert, 2010). Using Minitab ver.16 software, a cluster analysis was performed with an agglomerative hierarchical method to assess the relative variations of the different populations. The Euclidean method was used as a distance measure to create a dendrogram for clustering the populations.

**Table 1:** Populations of *Mesocriconema xenoplax* collected from different plants and localities used in the present study

**Population code**	**Life stage**	**Locality**	**Host**	**Accession numbers**	
				**D2-D3 rDNA**	***COI***
Mx1	Female	Fars province, Dokohak	Sour cherry	MT951888	MT951727
Mx2	Female	Fars province, Khan Zeniyan	Grapevine	MT951889	MT951728
Mx3	Female	Fars province, Dokohak	Grapevine	MT951890	MT951729
Mx4	Female	Fars province, Dasht Arjan	Almond	MT951891	MT951730
Mx5	Female	Fars province, Dokohak	Almond	MT951892	MT951731
Mx6	Female	Fars province, Dokohak	Apricot	MT951893	MT951732
Mx7	Female	Fars province, Sepidan	Apple	MT951894	MT951733
Mx8	Female	Fars province, Khan Zeniyan	Walnut	MT951895	MT951734
Mx9	Female	Fars province, Dasht Arjan	Walnut	MT951896	MT951735
Mx10	Female	Fars province, Dokohak	Walnut	MT951897	MT951736
Mx11	Female	Fars province, Ghasrodasht	Walnut	MT951898	MT951737
Mx12	Female	Fars province, Maharloo	Walnut	MT951899	MT951738
Mx13	Female	Fars province, Bajgah	Greengage	MT951900	MT951739
Mx14	Female	Fars province, Sepidan	Peach	MT951901	MT951740

### Phylogenetic relationships of the recovered populations

#### DNA extraction

A live female specimen of each population was picked out, examined on a microscopic slide and put in 8 μl ddH_2_O in a 0.2 microtube after photography. DNA was extracted according to Tanha Maafi et al. (2003) with some modifications: the tube was frozen at −80°C for at least 15 min and vortexed, and 10 μl worm lysis buffer (500 mM KCl, 100 mM Tris-Cl pH 8, 15 mM MgCl_2_, 0.05% Mercaptoethanol, and 4.5% Tween 20) and 2 μl of proteinase K (600 μg/ml) were added, respectively. The sample was incubated at 65°C for 1 h and at 95°C for 10 min. After incubation, the tube was centrifuged at 13000 rpm for 2 min and kept at −20°C for the next use.

#### PCR and sequencing

Two μl of the DNA template was added to the PCR reaction mixture containing 12 μl Master Mix RED (Ampliqon A/S, Denmark), 1 μl (10 pmol/μl) of each primer, to a final volume of 25 μl. The following primer sets were used for PCR: the forward D2A (5′-ACAAGTACCGTGAGGGAAAGTTG-3′) and the reverse D3B (5′-TCGGAAGGAACCAGCTACTA-3′) primers (De Ley et al., 1999) for amplification of the D2-D3 expansion segments of 28S rRNA gene, and COI-F5 (5′-AATWTWGGTGTTGGAACTTCTTGAAC-3′) and COI-R9 (5′-CTTAAAACATAATGRAAATGWGCWACW ACATAATAAGTATC-3′) primers for amplification of *COI* (Powers et al., 2014).

The following PCR amplification profiles were used for PCR reactions; D2-D3: 5 min at 94°C; 35 cycles of 30 seconds at 94°C, 45 seconds at 60°C and 1 min at 72°C, followed by a final step of 5 min at 72°C; *COI*: 5 min at 94°C; 35 cycles of 30 seconds at 94°C, 30 seconds at 48°C and 1 min at 72°C, followed by a final step of 5 min at 72°C. Four μl of each of the PCR products was run on a 1.5% Tris-borate-EDTA (TBE) buffered agarose gel (75 V, 45 min). The remaining PCR product was stored at −20°C. The PCR products were sequenced in both directions using the same primers with an ABI 3730XL sequencer (Bioneer Corporation, South Korea). Sequences were deposited into the GenBank database under accession numbers MT951888-MT951901 for 28S rRNA and MT951727-MT951740 for *COI* (Table 1).

#### Phylogenetic analyses

The newly obtained sequences were edited with Chromas 1.45 and aligned with the corresponding published gene sequences using Clustal X 1.64 (Thompson et al., 1997). The alignment program was run with default parameters (gap opening 15.0; gap extension 6.66). Outgroup taxa for *COI* were selected based on Powers et al. (2014) and for 28S rRNA, based on Subbotin et al. (2005). Sequence alignments were analyzed with Bayesian Inference (BI) using MrBayes 3.1.2 (Ronquist and Huelsenbeck 2003) under the GTR + G + I model. BI analysis for each gene was initiated with a random starting tree and was run with four chains for 2.0 × 10^6^ generations. Two runs were performed for each analysis. The Markov chains were sampled at intervals of 100 generations. After discarding burn-in samples (10%), a consensus tree with a 50% majority rule was generated. The posterior probabilities (PP) in percentages are given for the corresponding clades.

### Exploring genetic diversity based on ISSR markers

ISSR-PCR reactions were performed in a 20 μl volume containing 2 μl of genomic DNA, 2 μl of each primer, 8 μl of Master Mix RED (Ampliqon A/S, Denmark) and 8 μl of ddH_2_O. Seven primers, including (CCA)_5_, (GTG)_6_, (GAG)_4_GC, (GACA)_4_, (AC)_8_T, (CA)_8_G and (GAGA)_4_GG were used (Lax et al., 2007). PCR amplification reactions were programmed for initial denaturation at 94°C for 3 min, followed by 35 cycles of 30 s at 93°C, 90 s at 48°C, 1 min at 72°C, and a final extension of 10 min at 72°C. PCR success was visualized by running 7 μl of the PCR product on a 1.7% TBE buffered agarose gel (75 V, 120 min).

For ISSR fragment analysis, data were scored for all primers: “1” for the presence of the band and “0” for the absence of the band. Distance matrixes (D) were calculated using the Dice and Jaccard coefficients (Huff et al., 1993), performed by a Principal Coordinate Analysis (PCA) to obtain a graphical representation of the relationship between nematode populations. The basic population parameters (Nei, 1973; McDermott and McDonald, 1993) including polymorphism (P), heterozygosity (H), Shannon diversity (I), and Nei’s gene diversity (uHe) were estimated based on the banding pattern observed. The fixation index (F_st_) and indirect gene flow (Nm) were calculated based on formulae developed by Slatkin and Barton (1989).

A cluster analysis was performed using the Numerical Taxonomy Multivariate Analysis System (NTSYSPC V-2.02) software package (Rolf, 2000). We used the ISSR marker to create UPGMA and NJ trees. The genetic dissimilarity matrix and ultrametric distance matrix generated from a UPGMA-based dendrogram with a COPH module in the same software were compared with the Mantel matrix correspondence test (Mantel, 1967). The result of this test is a cophenetic correlation coefficient, *r*, indicating how well the dendrogram represents similar data. For the allelic frequency analyses, we defined the populations in two procedures: the first for each associated plant without considering the geographic regions and the second for the geographic regions at the level of a city without considering the associated plant. The allele frequency and analysis of molecular variance (AMOVA) were carried out using GenALEx version 6.5 (Peakall and Smouse, 2006).

## Results

### Morphological characterization of the recovered populations

**Figure 1:**
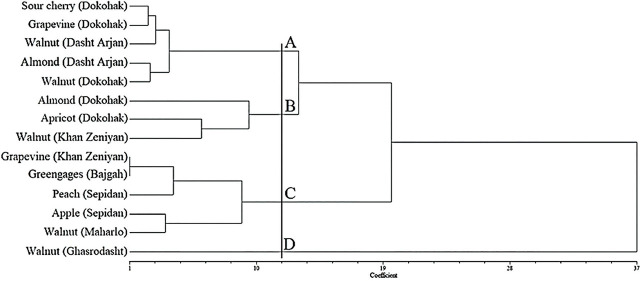
Similarity dendrogram of 14 populations of *Mesocriconema xenoplax* as compared by UPGMA analysis of morphological characters.

The recovered populations were characterized as *M. xenoplax* based on morphological and morphometric characteristics. The measurements of all recovered *M. xenoplax* populations are listed in Table S1 (see supplementary data). Cluster analysis identified four distinct groups labeled as A to D where A and B were closely related to each other and both were associated with group C, while a population recovered from the rhizosphere of walnut (Ghasrodasht) formed the last group D (Fig. 1).

### Phylogenetic relationships of the recovered populations

#### The inferred tree from the COI gene

**Figure 2:**
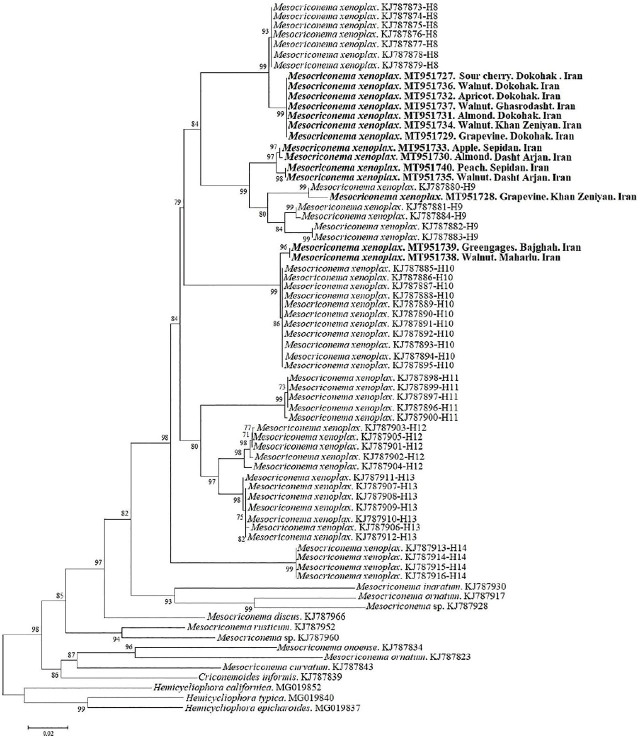
NJ tree of *COI* nucleotide sequences. New sequences are in bold (HG of Powers et al., 2014 are written in the front of sequences of *Mesocriconema xenoplax*).

The *COI* gene alignment was 681 bp long and consisted of 68 sequences as ingroups and three sequences of *Hemicycliophora californica* Brzeski, 1974, *H. typica* de Man, 1921 and *H. epicharoides* Loof, 1968 as outgroups. Fourteen new sequences of the *COI* gene were obtained in the present study. For phylogenetic analyses, 58 sequences of *M. xenoplax* from Powers et al. (2014) were included here to investigate the phylogenetic relationships of Iranian populations with known HG. The phylogenetic relationships of newly sequenced isolates with other populations of *M. xenoplax* with collapsed branches (PP less than 70%) are shown in Fig. 2. Seven HG (HG8-14) introduced by Powers et al. (2014) were observed in our analyses as separate clades. In general, presently studied populations occupied four positions in the tree: *i*) seven populations, including those associated with sour cherry (Dokohak), walnut (three isolates from Dokohak, Ghasrodasht and Khan Zeniyan), apricot (Dokohak), almond (Dokohak), and grapevine (Dokohak) formed a sister group with HG8 sensu Powers et al. (2014), differing by only three nucleotides (0.44% nucleotide divergence); *ii*) the population associated with grapevine (Khan Zeniyan) was placed among the sequences of HG9 sensu Powers et al. (2014), especially close to a population from Wakulla County-USA; however, these two sequences differed by six nucleotides (0.88% nucleotide divergence); *iii*) two populations associated with apple and peach (Sepidan) and two other populations from almond and walnut (Arjan) formed a relatively separate lineage related to HG9 sensu Powers et al. (2014) but with nucleotide diversity at 34 sites (4.99%) compared to the sequences of HG9 sensu Powers et al. (2014); and *iv*) two populations of greengage (Bajgah) and walnut (Maharloo) were placed with sequences of HG10 sensu Powers et al. (2014) with only one nucleotide (0.14%) difference.

### The tree inferred from D2-D3 expansions of the 28S rRNA gene

**Figure 3:**
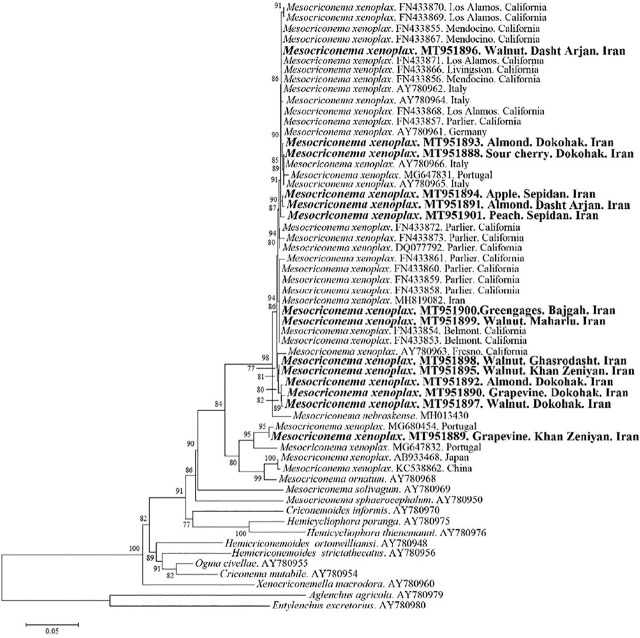
Bayesian Inference (BI) tree of D2-D3 expansion segments of 28S rRNA nucleotide sequences. New sequences are in bold.

The D2-D3 alignment was 541 bp long and consisted of 56 sequences as ingroups and two sequences, including *Aglenchus agricola* (de Man, 1884) Andrassy, 1954 and *Eutylenchus excretorius* Ebsary and Eveleigh, 1981 as outgroups. Fourteen new sequences of the D2-D3 expansion fragments of the 28S rRNA gene were obtained in the present study. The phylogenetic relationships of the Iranian populations within other populations of *M. xenoplax* and certain closely related species, inferred from the analysis of these partial 28S rRNA gene sequences with collapsed branches, with a PP of less than 70%, are shown in Fig. 3.

Nucleotide diversity within the Iranian populations varied between zero and 61 nucleotides (0–11.46%). It seems that two distinct lineages could be considered for populations of *M. xenoplax*. A large proportion of the populations of *M. xenoplax* with 13 Iranian populations of this species formed one lineage (*M. nebraskense* showed high affinity with this lineage) and the grapevine-Khan Zeniyan population formed another lineage with two populations from Portugal, one population from Japan, and one population from China (one population of *M. ornatum* with close affinity to this lineage).

### Genetic diversity among populations based on ISSR markers

**Figure 4:**
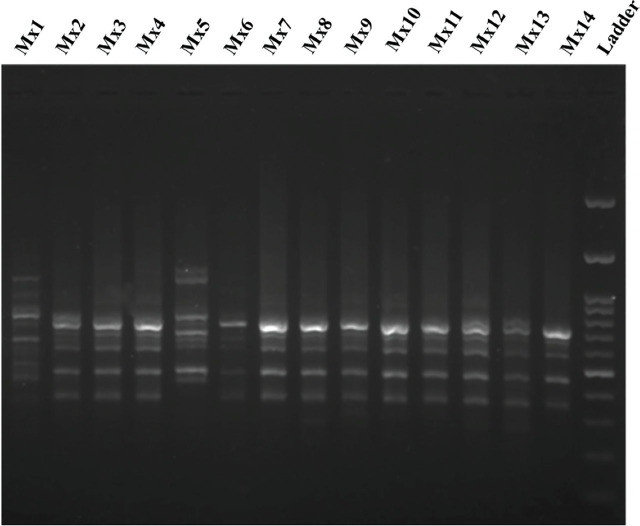
ISSR patterns for 14 populations of *Mesocriconema xenoplax* generated with the primer (CA)_8_G. DNA Ladder = 200 bp.

A total of 89 loci with an average of 12.7 loci per primer were scored with the ISSR marker. ISSR patterns for 14 populations of *M. xenoplax* generated with primer (CA)_8_G are shown in Fig. 4 (data not shown for other primers).

Considering the populations by associated plants, the mean genetic variations of the total indicated by P, I, H and uHe were 6.18%, 0.039, 0.027 and 0.033, respectively, indicating a very low genetic diversity. Since only one population each from sour cherry, apricot, apple, greengage and peach was collected, values of P, I, H and uHe could not be calculated for these populations. However, after excluding these populations, five walnut populations exhibited the highest genetic diversity (P% = 23.60, I = 0.154, H = 0.108 and uHe = 0.121). It seems that there is no correlation between population size and genetic diversity, as two populations were evaluated for both grapevine and almond, but the genetic diversity of the two almond populations (P% = 19.10, I = 0.116, H = 0.079 and uHe = 0.105) was much higher than that of grapevine (P% = 6.74, I = 0.041, H = 0.028 and uHe = 0.037), almost close to the walnut populations.

For the second comparison based on regions, the populations of Ghasrodasht, Maharloo and Bajghah were excluded as they had only one population. The populations from Dokohak and Khan Zeniyan showed the highest genetic diversity (P% = 34.83, I = 0.231, H = 0.164, uHe = 0.182 and P% = 21.35, I = 0.129, H = 0.088, uHe = 0.118, respectively), while the lowest value was found in the populations from Sepidan (P% = 4.49, I = 0.027, H = 0.019 and uHe = 0.025). The mean total genetic variation indicated by P, I, H and uHe, were 10.91%, 0.069, 0.048 and 0.059, respectively, indicating low genetic diversity.

The genetic distances (D) between pairs of *M. xenoplax* populations by associated plants determined by Nei (1978) varied from 0.034 to 0.269. The lowest value was between populations of sour cherry and apricot, and the highest between populations of grapevine and apricot. In the ISSR analysis by geographic region, pairwise D values varied from 0.082 (for the populations of Dokohak and Khan Zeniyan) to 0.315 (for the populations of Sepidan and Ghasrodasht) (Table 2).

**Table 2:** Nei’s unbiased genetic distance between pairs of the *Mesocriconema xenoplax* populations from Fars province, Southern Iran, detected by ISSR analyses.

**Associated plants**	**Sour cherry**	**Apricot**	**Walnut**	**Grapevine**	**Almond**	**Apple**	**Greengage**	**Peach**
Sour cherry	***	-	-	-	-	-	-	-
Apricot	0.034	***	-	-	-	-	-	-
Walnut	0.151	0.194	***	-	-	-	-	-
Grapevine	0.225	0.269	0.196	***	-	-	-	-
Almond	0.208	0.252	0.099	0.086	***	-	-	-
Apple	0.171	0.212	0.165	0.205	0.167	***	-	-
Greengage	0.185	0.226	0.161	0.239	0.181	0.058	***	-
Peach	0.158	0.198	0.179	0.239	0.181	0.034	0.070	***

**Geographic regions**	**Dokohak**	**Khan Zeniyan**	**Dasht Arjan**	**Sepidan**	**Ghasrodasht**	**Maharloo**	**Bajgah**
Dokohak	***	-	-	-	-	-	-
Khan Zeniyan	0.082	***	-	-	-	-	-
Dasht Arjan	0.100	0.111	***	-	-	-	-
Sepidan	0.170	0.200	0.181	***	-	-	-
Ghasrodasht	0.191	0.229	0.245	0.315	***	-	-
Maharloo	0.208	0.200	0.167	0.242	0.284	***	-
Bajgah	0.248	0.264	0.270	0.143	0.411	0.314	***

**Figure 5:**
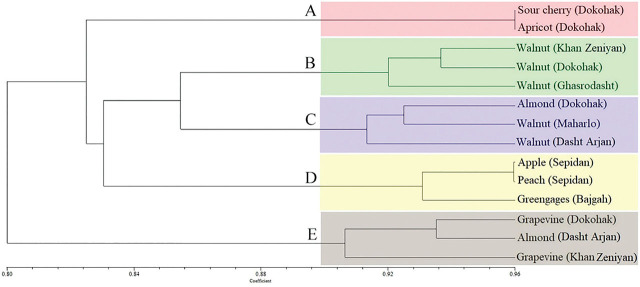
UPGMA cluster diagram based on Dice coefficient estimated from 89 ISSR markers for 14 populations of the *Mesocriconema xenoplax* (host plants and geographic regions are written in the front of branches).

**Figure 6:**
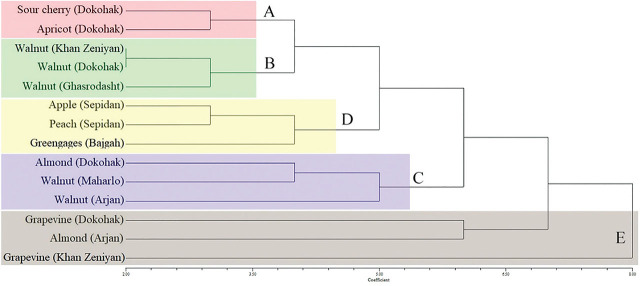
NJ cluster diagram from 89 ISSR markers for 14 populations of the *Mesocriconema xenoplax* (host plants and geographic regions are written in the front of branches).

**Figure 7:**
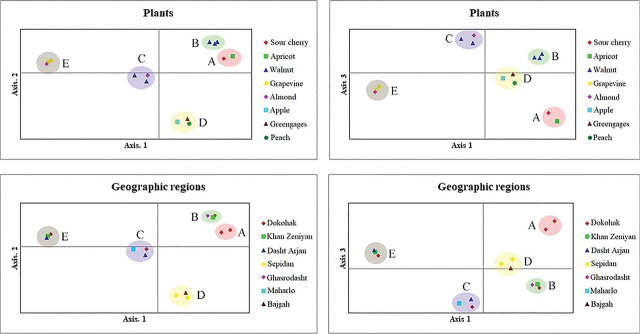
The bi-dimensional dispersion according to the principal coordinates analysis (PC1 *vs* PC2 and PC1 *vs* PC3) obtained using the GenAlex software for *Mesocriconema xenoplax* populations from Fars province, Southern Iran by ISSR analyses (above: populations defined by plants, below: populations defined by geographic regions).

The UPGMA and NJ trees obtained from the ISSR data were presented in Fig. 5 and Fig. 6, respectively. For each tree, five different groups of A-E were obtained. The trees were generally very similar and only some minor differences were found in the placement of certain groups and populations. The main differences were the separation of the grapevine population (Khan Zeniyan) and the placement of this population in a separate line in the NJ tree (Fig. 6). However, this population formed a well-separated clade in the UPGMA tree with the grapevine (Dokohak) and almond (Arjan) (Fig. 5). In addition, groups A and B were closely related to group C in the UPGMA tree, but these two groups were closer to group D than to group C in the NJ tree. PCA plots for PC1 *vs.* PC2 and PC1 *vs.* PC3 based on plants and geographic regions were shown in Fig 7. In the PCA plots, similar five groups of UPGMA and NJ trees were highlighted. The two principal coordinates (PCs) were accumulated at 64% and 68%, and the three PCs were accumulated at 78% and 81% for plants and geographic regions, respectively.

The percentage of molecular variance (AMOVA) among and within populations was 61% and 39% for the populations grouped by plant, and 50% when grouped by geographic region. The values of F_ST_ and Nm by plant were 0.611 (P = 0.010) and 0.159, respectively, and by geographic region were 0.504 (P = 0.010) and 0.246, respectively (Table 3). The values of F_ST_ and Nm among populations of the different plants are shown in Table 4. For a large proportion of the plant relationships, a good separation was obtained among the populations of the individual plants and the other plants (F_ST_ > 0.800 and Nm < 0.100). The highest F_ST_ value (and consequently the lowest value of Nm) was obtained between apricot and apple (F_ST_ = 0.911 and Nm = 0.024), while the F_ST_ value between walnut and almond, and grapevine and almond was very low (0.103 and 0.120, respectively), indicating a very high gene flow between nematode populations associated with these plants (Nm was 2.177 and 1.833, respectively).

**Table 3:** Analysis of molecular variance (AMOVA) of the *Mesocriconema xenoplax* populations, based on ISSR data

**Type of Population**	**Source of variation**	**Degree of freedom**	**Sum of squares**	**Variance components**	**Percentage of variation**	**(*F_ST)_***	**(Nm)**
Host	Between populations	7	94.205	4.559^*^	61	0.611	0.159
	Within populations	7	31.900	2.900^*^	39		
	Total	14	126.105	7.459			
Geography	Between populations	6	98.794	4.937^*^	50	0.504	0.246
	Within populations	8	48.500	4.850^*^	50		
	Total	14	147.294	9.787			

* P<0.001.

The values of F_ST_ and Nm among populations of different geographical regions were also determined (Table 4). The F_ST_ values among populations from different geographic regions were lower than those for among plants (F_ST_ < 0.400), and a moderate to strong gene flow occurred among populations of the different geographic regions (Nm > 0.400). The highest F_ST_ value and consequently the lowest Nm value was found for Arjan-Bajgah (F_ST_ = 0.382 and Nm = 0.404) and minimum F_ST_ value (0.111) for Khan Zeniyan-Arjan (Nm = 2.002).

**Table 4:** Pairwise estimates of *F_ST_* and *Nm* among populations of *Mesocriconema xenoplax* recovered from Fars province, Southern Iran by ISSR analyses.

**Plants**	**Sour cherry**	**Apricot**	**Walnut**	**Grapevine**	**Almond**	**Apple**	**Greengage**	**Peach**
Sour cherry	***	0.032	0.306	0.050	0.236	0.030	0.027	0.039
Apricot	0.887	***	0.211	0.042	0.177	0.024	0.030	0.033
Walnut	0.450	0.542	***	0.288	2.177	0.259	0.253	0.230
Grapevine	0.833	0.857	0.465	***	1.833	0.053	0.047	0.047
Almond	0.514	0.585	0.103	0.120	***	0.303	0.265	0.265
Apple	0.893	0.911	0.491	0.824	0.452	***	0.038	0.031
Greengage	0.902	0.892	0.497	0.842	0.485	0.869	***	0.027
Peach	0.865	0.884	0.521	0.842	0.485	0.891	0.903	***

**Geographic regions**	**Dokohak**	**Khan Zeniyan**	**Dasht Arjan**	**Sepidan**	**Ghasrodasht**	**Maharloo**	**Bajgah**
Dokohak	***	1.549	1.462	0.679	0.697	0.662	0.606
Khan Zeniyan	0.139	***	2.002	0.443	0.719	0.924	0.649
Dasht Arjan	0.146	0.111	***	0.800	0.637	0.618	0.404
Sepidan	0.269	0.361	0.238	***	0.539	0.583	0.501
Ghasrodasht	0.264	0.258	0.282	0.317	***	0.828	0.935
Maharloo	0.274	0.213	0.288	0.300	0.232	***	0.524
Bajgah	0.292	0.278	0.382	0.333	0.211	0.323	***

*F_ST_* (below diagonal) and Nm (above diagonal).

## Discussion

In this study, 14 populations of *Mesocriconema xenoplax* from orchards in Fars province, Southern Iran, were analyzed for morphological, phylogenetic, and genetic characteristics. We used reconstructed phylogenetic trees based on 28 new sequences of *COI* and 28S rRNA genes, and ISSR-based markers to estimate the intraspecific variation in this nematode species. This integrative study of different populations of this species can improve our understanding of the taxonomic intra-generic structure of *M. xenoplax* (as a representative of Criconematidae) and provides information on the genetic diversity of this cosmopolitan species.

Different populations of *M. xenoplax* from North America formed a monophyletic group consisting of seven genetically distinct *COI* subgroups (Powers et al., 2014). The *COI* sequences revealed a close relationship between the Iranian populations of *M. xenoplax* and HG of 8 to 10 from North America. Powers et al. (2014) reported high nucleotide diversity for HG9 (0.02469) compared to other HGs. The authors, moreover, discussed that this HG may be subdivided into some HGs by including additional sequences. Confirming this, four populations in the present study created a relatively separate lineage related to HG9 and they may belong to other new HG.

In addition, the phylogenetic 28S rRNA tree revealed two distinct lineages for all known isolates of this species. While most isolates from Iran and other localities formed a major clade together, one of our isolates from grapevine (Khan Zeniyan) together with some other isolates from Portugal, Japan, and China formed another basal clade to this major lineage. However, this population showed no differences in terms of morphological characters and formed Group C in the similarity dendrogram with other populations from other plants based on morphological characters (Fig. 1).

ISSR markers revealed low genetic diversity between nematode populations recovered from host trees and geographic regions in the present study; however, the estimated nematode genetic diversity among geographic regions was slightly higher than that in the associated plant trees (5.9% and 3.3%, respectively). Values higher than 0.25 for Nei’s index could be interpreted as very high genetic diversity among populations (Zhou et al., 2016). The Nei’s index was below 0.25 for most of the associated plants in the present study, and only populations of grapevine showed values slightly higher than 0.25 with other plants (0.252 and 0.269 for grapevine-almond and grapevine-apricot, respectively). In terms of geographical locations, the population from Bajgah showed higher values than 0.25 for all other regions except Sepidan, where the Nei index was lower (0.143), indicating genetic variation between the Bajgah population and most other populations.

In general, the results of our study based on ISSR markers suggested adequate gene exchange between different populations of *M. xenoplax* recovered from various plants and localities (confirmed by population parameters such as F_st_, Nm, P%, I, and uHe), leading to very low genetic diversity (confirmed by low values of Nei’s index). However, certain populations (e.g., grapevine from Khan Zeniyan) showed a higher level of genetic distance and mostly occupied distinct separate clade in the phylogenetic trees or ISSR clustering diagrams. However, it did not occupy a distant position in the similarity dendrogram based on morphological characteristics and moreover, morphological or morphometric data did not reveal any significant difference between this population and other populations. Therefore, our results showed that there is a low level of gene flow among certain populations, but the amount of total gene flow was high between most of the populations. Similar results have been already observed for *M. xenoplax* (Powers et al., 2014) and *Meloidogyne enterolobii* (Shao et al., 2020) in which total genetic diversity of the nematode species is low (high amount of gene flow is observed among populations), but certain populations show different degrees of genetic variability.

In addition, we found that the genetic variation of *M. xenoplax* comes from both within and between populations sources. However, different populations from the same plant/geographic region usually placed in distinct clades in the ISSR diagrams, but obvious genetic structure driven by plant or geographic region was rarely observed. For instance, two grapevine populations as well as two populations from Sepidan placed in the same group. However, our study lacks enough replications for most of the plants or geographic regions to capture the full extent of variability within and between groups. Therefore, future works with a higher number of populations may provide additional information on the genetic diversity of the nematode.

The low genetic diversity observed in *M. xenoplax* in the present study could be attributed to the parthenogenetic mode of reproduction. Similarly, some other researchers (Triantaphyllou, 1985; Tigano et al., 2010; Ghaderi et al., 2020) considered parthenogenesis as a main factor responsible for low genetic diversity in other nematode species. However, genetic diversity was even lower than in another parthenogenetic species, *Meloidogyne javanica*, which was more than 8% in different populations from Iran (Ghaderi et al., 2020; Mokaram Hesar et al., 2022).

The most important factors for the spread of nematodes over long distances are human activities and transferring of plant material such as seedlings (Been and Schomaker, 2013; Powers et al., 2014). It may be concluded that such types of nematode dispersal have occurred in the studied region, leading to greater genetic uniformity. Based on haplotype analysis of North American populations, Powers et al. (2014) confirmed the monophyletic nature of *M. xenoplax*; however, they noticed that deep divisions in the clade of this species may provide evidence for both geographic localization and long-distance dispersal associated with agricultural practices. In summary, it should be noted that other approaches like specific analysis of the *COI* mitochondrial gene for defining HG (Powers et al., 2014; Shao et al., 2020) or genotyping by sequencing (Rashidifard et al., 2018) can obtain more detailed information and present a better understanding of the *M. xenoplax* genetic diversity.

## Supplementary Data

**Table S1:** Morphometric characters of the *Mesocriconema xenoplax* populations recovered from the rhizosphere of different plants and localities in Fars province, Southern Iran (measurements are in μm for 10 females per plant).

**Characters**	**Almond**	**Almond**	**Apple**	**Apricot**	**Grapevine**	**Grapevine**	**Greengage**	**Peach**	**Sour Cherry**	**Walnut**	**Walnut**	**Walnut**	**Walnut**	**Walnut**	**Total**	

	**Dasht Arjan**	**Dokohak**	**Sepidan**	**Dokouhak**	**Khan Zeniyan**	**Dokohak**	**Bajgah**	**Sepidan**	**Dokohak**	**Dasht Arjan**	**Dokohak**	**Ghasrodasht**	**Maharlu**	**Khan Zeniyan**	**140 ♀**	**CV**
L	547–712	595–762	510–610	533–679	481–649	541–667	488–631	446–598	490–676	530–629	543–720	661–787	479–619	561–720	594 ± 68 (446–787)	11.5
a	9.5–12.4	10.7–13.8	10.9–13.6	10.3–12.0	8.7–11.6	9.8–12.2	8.7–11.6	8.9–11.8	7.9–15.2	9.7–15.3	9.3–13.4	10.3–12.9	8.7–12.1	10.0–16.5	11.2 ± 1.3 (7.9–16.5)	11.8
b	3.5–5.0	4.2–5.1	3.8–4.5	3.9–4.9	3.5–4.4	3.7–4.5	3.5–4.5	3.6–4.2	3.7–4.9	3.7–4.6	3.7–4.7	3.9–5.6	3.2–4.8	4.0–5.1	4.2 ± 0.4 (3.2–5.6)	10.0
c	25.1–50.0	27.2–62.5	28.3–46.7	36.5–54.8	24.3–49.5	29.6–51.3	26.0–44.1	27.8–42.8	31.8–43.3	32.3–49.9	36.7–63.2	38.1–54.5	29.6–51.2	33.2–52.1	39.3 ± 7.9 (24.3–63.2)	20.1
V	93.5–95.4	93.8–95.7	93.4–94.6	94.7–95.8	92.1–94.4	94.6–96.0	91.9–94.5	93.5–96.0	94.5–95.4	94.6–95.7	94.6–95.9	93.9–95.5	94.0–95.4	93.3–95.7	94.6 ± 0.9 (91.9–96.0)	0.9
Stylet	69.3–77.5	67.0–76.5	74.0–79.0	65.4–74.7	70.8–80.0	66.1–73.1	70.8–80.0	66.0–76.2	57.6–78.3	70.5–79.8	70.0–79.7	74.6–79.8	57.0–77.3	69.0–81.3	73.5 ± 4.3 (57.0–81.3)	5.9
DGO	6.3–8.6	6.0–8.3	-	7.1–8.6	4.0–7.8	7.0–8.3	5.2–7.2	-	6.1–7.2	5.5–6.1	6.5–8.6	6.2–10.8	6.1–9.5	6.3–8.1	7.2 ± 0.9 (4.0–10.8)	12.2
Oesophagus	133–156	137–159	127–141	132–162	125–147	126–156	127–144	124–143	125–153	132–156	137–153	131–169	128–152	138–170	142 ± 9.1 (124–170)	6.4
S-E pore	135–171	146–183	134–158	142–182	128–163	135–166	131–152	132–151	138–173	140–169	137–177	146–203	133–163	149–176	154 ± 13.4 (128–203)	8.7
Head to vulva	515–679	566–729	477–574	506–643	447–606	514–635	455–587	424–566	466–639	503–597	513–690	620–750	450–585	534–671	561 ± 67 (424–750)	11.9
Tail	12.2–24.7	12.2–23.0	12.0–20.0	10.2–16.0	13.1–20.7	11.2–20.0	14.3–19.5	13.0–19.0	13.8–18.1	12.6–17.5	11.4–16.0	12.8–19.3	11.3–19.3	11.6–20.5	15.5 ± 2.5 (10.2–24.7)	15.9
Body width	48.0–59.5	51.2–57.5	38.0–55.0	45.2–60.7	45.6–55.8	52.4–58.4	46.3–56.2	45.0–55.0	43.3–62.0	41.0–61.4	49.7–62.3	56.0–68.7	44.0–63.0	43.7–64.3	53.3 ± 5.7 (38.0–68.7)	10.6
R	89–105	99–104	98–106	98–103	95–104	95–104	96–104	79–101	90–104	97–107	97–104	97–106	90–104	97–105	98 ± 5.4 (79–107)	5.5
Rst	10–17	9–14	15–17	9–15	14–17	9–15	14–17	11–15	11–15	13–16	13–16	11–15	12–17	12–15	14 ± 1.9 (9–17)	13.3
Roes	21–32	22–25	23–27	22–26	25–28	19–27	25–28	21–25	21–28	23–28	23–27	20–28	24–30	22–28	24.8 ± 2.2 (19–32)	8.8
Rex	24–33	24–28	26–29	24–29	25–30	21–28	25–30	22–27	25–30	25–30	25–29	24–28	25–31	24–29	26.9 ± 1.9 (21–33)	7.1
RV	6–8	6–9	6–7	5–7	7–9	5–7	7–9	5–6	4–6	5–6	5–6	6–8	5–6	5–7	6.2 ± 1.0 (4–9)	15.9
Ran	3–5	3–4	3–4	2–4	3–4	3–4	3–4	2–3	2–3	3–4	2–3	3–4	2–4	2–3	3.1 ± 0.6 (2–5)	18.4
Rvan	3	2–5	2–3	2–3	4–5	2–4	4–5	2–3	2–3	2–3	2–3	3–4	2–3	3–4	3.1 ± 0.8 (2–5)	25.0
VL	30.2–36.6	28.6–38.7	30.0–37.0	25.2–36.0	30.3–46.6	23.0–32.0	30.0–47.1	20.0–36.0	24.0–36.3	26.4–31.4	26.0–32.0	32.5–43.8	26.5–34.0	26.2–48.6	32.1 ± 4.8 (20.0–48.6)	14.9
VB	24.7–37.8	31.2–40.2	31.0–37.0	31.0–41.5	30.1–40.0	33.0–39.5	30.0–40.2	28.0–38.0	32.3–37.0	26.6–38.8	31.1–40.8	31.4–47.6	33.4–39.5	29.8–42.8	35.8 ± 3.6 (24.7–47.6)	10.1
VL/VB	0.9–1.5	0.8–1.0	0.9–1.2	0.7–0.9	0.8–1.2	0.7–1.0	0.8–1.2	0.7–1.0	0.7–1.0	0.8–1.2	0.7–0.9	0.7–1.3	0.7–0.9	0.7–1.2	0.9 ± 0.1 (0.7–1.5)	14.9
St/L %	10.7–13.5	9.6–12.3	12.6–15.1	9.6–14.0	11.9–16.6	10.5–13.0	12.2–16.3	12.6–15.0	9.6–13.5	11.2–15.0	10.4–13.7	10.1–11.7	9.9–15.3	10.3–12.9	12.5 ± 1.5 (9.6–16.6)	12.0
St/Oes %	45.6–57.5	45.9–51.6	54.6–61.4	43.8–55.3	49.2–60.8	44.3–55.8	49.2–59.8	50.4–56.4	43.0–55.8	49.2–60.5	48.8–55.7	45.8–56.9	40.4–56.0	47.8–53.9	51.7 ± 3.9 (40.4–61.4)	7.6
S-E pore/L %	24–30	21–28	23–28	24–29	22–31	23–29	23–30	25–30	23–30	24–28	24–28	22–27	22–33	23–30	26 ± 2 (21–33)	7.6

Abbreviations in the Table. L: body length; a: body length to the greatest body diameter; b: body length to oesophagus length; c: body length to tail length; V: vulva position from anterior end to body length (expressed as a percentage); stylet: length of stylet; DGO: pharyngeal dorsal gland orifice from stylet knobs; Oes: oesophagus length; S-E pore: the distance of secretory-excretory pore from anterior end; tail: length of tail; BW: greatest body diameter; R: total number of body cuticular annuli; Rst: number of cuticular annuli from anterior end to the base of stylet knobs; Roes: the number of cuticular annuli from anterior end to the end of oesophagus; Rex: number of cuticular annuli from anterior end to secretory-excretory pore; RV: number of cuticular annuli between posterior end of body to vulva; Ran: number of cuticular annuli posterior to anus; Rvan: number of cuticular annuli between vulva and anus; VL/VB: post-vulval region length to vulval body diameter; St/L: stylet length to body length (as a percentage); St/Oes: stylet length to oesophagus length (as a percentage); S-E pore/L: distance between anterior end and secretory-excretory pore to body length (as a percentage).
